# Variational model for a rippled graphene sheet

**DOI:** 10.1039/c9ra10439a

**Published:** 2020-04-22

**Authors:** Jabr Aljedani, Michael J. Chen, Barry J. Cox

**Affiliations:** School of Mathematical Sciences, University of Adelaide Adelaide Australia Jabr.aljedani@adelaide.edu.au; Faculty of Applied Studies, King Abdulaziz University Jeddah Saudi Arabia

## Abstract

The calculus of variations is utilised to study the behaviour of a rippled graphene sheet supported on a metal substrate. We propose a model that is underpinned by two key parameters, the bending rigidity of graphene *γ*, and the van der Waals interaction strength *ξ*. Three cases are considered, each of which addresses a specific configuration of a rippled graphene sheet located on a flat substrate. The transitional case assumes that both the graphene sheet length and substrate length are constrained. The substrate constrained case assumes only the substrate has a constrained length. Finally, the graphene constrained case assumes only the length of the graphene sheet is constrained. Numerical results are presented for each case, and the interpretation of these results demonstrates a continuous relationship between the total energy per unit length and the substrate length, that incorporates all three configurations. The present model is in excellent agreement with earlier results of molecular dynamics (MD) simulations in predicting the profiles of graphene ripples.

## Abstract 2

A variational model is proposed to describe rippled graphene on a substrate, and obtain the relationship between total energy and substrate length.

## Graphical abstract

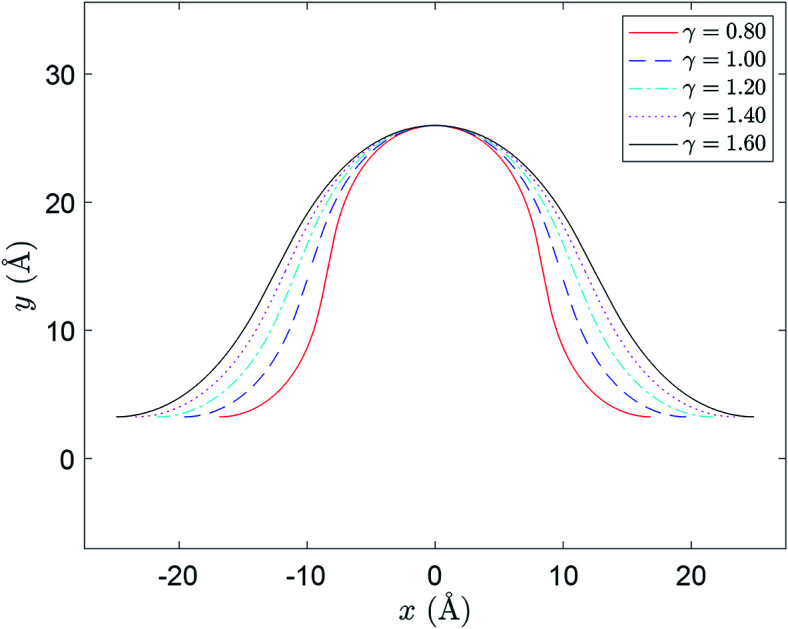

## 1 Introduction

Graphene is a two-dimensional sheet of carbon atoms bonded to each other in a planar hexagonal array. This two-dimensional structure endows graphene with many useful electronic and mechanical properties.^1–7^ These properties mean graphene is a highly promising material for constructing nanoelectromechanical systems.^8,9^ Graphene is hypothesised to be a biocompatible material since its bending stiffness is comparable with that of the lipid bilayers of the biological cells,^10^ and it has many other applications in gas separation,^11^ biomedicine,^12^ nitrogen reduction reaction,^13^ and metal-ion batteries.^14^ For example, popgraphene is a beneficial anode material in lithium-ion batteries with fast charge and discharge rates.^15^

However, graphene does not always remain planar. For example, ripples are observed in suspended graphene sheets,^16^ as well as graphene on a substrate.^17^ Experimental studies find that the electronic properties of graphene can be affected by the range and height of these ripples.^18,19^ Gui *et al.*^20^ use first-principles calculations to predict the electronic properties of a rippled graphene sheet where a band gap opening is observed in the rippled graphene. They evaluate a direct band gap at 0.93 eV which indicates rippled graphene may be a highly tunable semiconductor. They also report that the band gap increases proportionally with ripple amplitude, which is the maximum distance between the graphene sheet and the substrate.

The pattern of ripples in graphene may be affected by a variety of factors, including temperature, substrate material, and the size of the graphene sheet. For example, suspended graphene remains flat with no ripples when the temperature is close to absolute zero, but ripples appear as the temperature increases.^21^ When a graphene sheet is placed on a substrate the amplitude of the ripples depends on the substrate properties such as roughness and interfacial van der Waals interactions.^17^ Moreover, the overall size of the graphene sheet has a significant impact on the amplitude of ripples, with the ripple amplitude increasing proportionally with the size of the sheet.^22^

The bending rigidity of graphene plays an important role in the conformation of ripples. Using density functional theory calculations, Wei *et al.*^10^ evaluate the bending rigidity of a single-layer graphene at 1.44 eV. They compare this result to earlier obtained values for the bending rigidity of graphene which range from 0.80 to 1.60 eV. To cover this range of values, we will consider various bending rigidities for the rippled graphene in the present paper.

The energies we consider when modelling ripples in graphene are the same as those considered when modelling a fold of graphene. Cox *et al.*^23^ develop a model which describes a graphene sheet folded onto itself where two energies are taken into account. The first is the elastic energy that arises when bending a graphene sheet which is represented mathematically by integrating the square of the line curvature over the length of the total curve and scaled by the bending rigidity of graphene. The second energy is the van der Waals (vdW) interaction between the flat section of the graphene layers, prescribed as the integral of a Heaviside unit step function multiplied by the vdW energy per unit area. An extension of the fold model was further developed by Dyer *et al.*^24^ where either a carbon nanotube of circular or elliptical cross section is encapsulated in the graphene fold. A comparison between MD simulations and the mathematical model shows excellent agreement between the two approaches in determining the fold shape.

In the following section, we formulate a general mathematical model of a single graphene ripple on a substrate. In Section 3 we non-dimensionalise this model and employ the calculus of variations assuming a fixed length (isoperimetric) constraint of both the rippled graphene sheet and the substrate. In Section 4 we relax the isoperimetric constraint on the sheet length, but maintain a fixed substrate length. The substrate length is varied in Section 5 and an isoperimetric constraint is applied to only the sheet length. The solutions and associated numerical details are provided with each case. The main results and the relationships between the three cases considered here are described in Section 6. Discussion and some concluding remarks are made in Section 7.

## 2 Model formulation

**Fig. 1:**
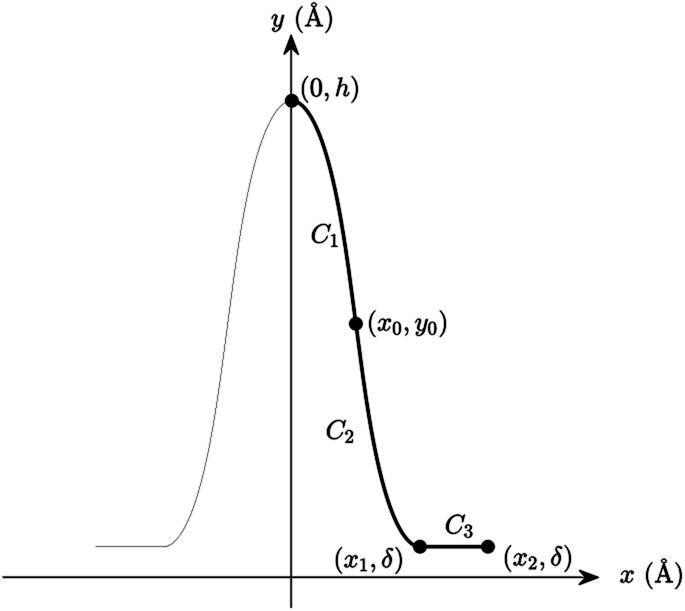
Geometry of the rippled graphene.

We propose a general formulation to determine the conformation of a rippled sheet of graphene on a substrate for each of the three cases mentioned above. We consider two different metal substrates: Cu(111) and Ni(111). The geometry of the rippled graphene sheet is shown in Fig. 1. Here we assume a translational symmetry in the *z*-direction which reduces the problem to two dimensions. Also, the reflective symmetry about the *y*-axis allows us to consider only solutions in the right half plane. We further divide the solution curve into three sections. The first section *C*_1_ is the curve from the point (0, *h*) to the point (*x*_0_, *y*_0_) where the line curvature is strictly negative. The second section *C*_2_ is from (*x*_0_, *y*_0_) to (*x*_1_, *δ*) where the line curvature is strictly positive. The third section *C*_3_ is the horizontal line from (*x*_1_, *δ*) to (*x*_2_, *δ*) where the line curvature is zero. The concatenation of these sections is denoted by *C* = *C*_1_ + *C*_2_ + *C*_3_. In these position vectors we use *h* to denote the ripple height, and *δ* to denote the separation distance between the substrate and the flat section of the graphene sheet.

With reference to the model developed by Cox *et al.*,^23^ the dominant energies for our model are the elastic bending energy and the vdW interaction energy. The elastic energy *E*_e_ is modelled with
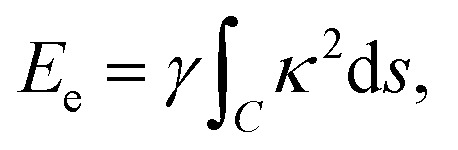
where *γ* is the bending rigidity of graphene, *s* is the arc length, and *κ* is the line curvature of *y* = *y*(*x*) which is given by
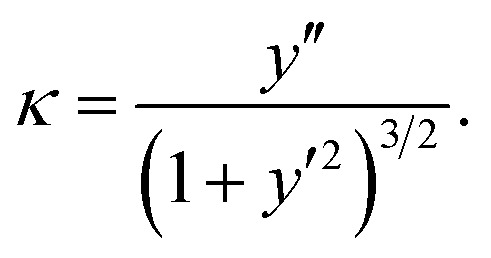


As mentioned in Section 1, the bending rigidity of graphene reported by various authors ranges from 0.8 to 1.6 eV as presented in ref. 10, and for the purpose of comparison we consider a linear sample from this range for *γ*, namely, 0.8, 1.0, 1.2, 1.4, and 1.6 eV. We model the vdW interaction energy between the graphene and the substrate as
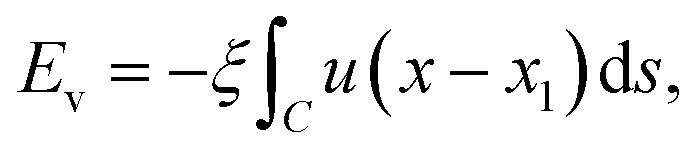
where *ξ* denotes the vdW energy of graphene–substrate interactions per unit area, and *u*(*x* − *x*_1_) is a Heaviside unit step function. Our model assumes that the elastic energy dominates in sections *C*_1_ and *C*_2_, and the vdW interaction dominates in *C*_3_. We comment that the curvature and therefore the elastic energy vanishes in *C*_3_, but discarding the vdW interaction in sections *C*_1_ and *C*_2_ is an approximation we make in the modelling. Therefore, the total energy per unit length may be expressed by1
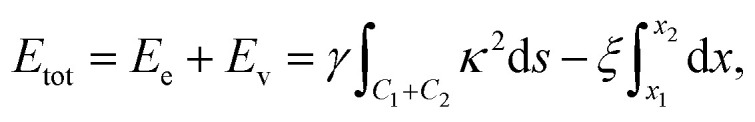
subject to the boundary conditions

2 *x*(0) = 0, *y*(0) = *h*, *ẏ*(0) = 0, *y*(*x*_1_) = *δ*, and *ẏ*(*x*_1_) = 0

where at the endpoint *x* = *x*_1_ the value of *x*_1_ is not prescribed and we have a natural boundary condition on *x*. Here dots denote differentiation with respect to *x*.

**Fig. 2:**
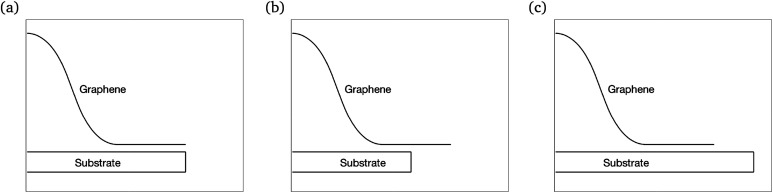
Schematic showing the geometries of: (a) the transitional case, (b) the substrate constrained case, and (c) the graphene constrained case.

For the purpose of comparison we prescribe a particular value of *h* for each case of a rippled graphene sheet supported on a different substrate, and the reason for adopting these particular values will be explained in Section 4. The prescribed ripple height, *h*, and empirical values for the separation distance between graphene and the substrate, *δ*, and the vdW energy of graphene–substrate interactions per unit area, *ξ*, corresponding to each substrate are shown in Table 1. Now we apply this model to consider the three cases for the length of graphene sheet placed on a substrate, namely, the transitional case, the substrate constrained case, and the graphene constrained case. Fig. 2 illustrates the geometry of each case.

**Table 1:** Empirical values (with the relevant references given in superscript) for *δ*, *ξ*, and the prescribed ripple height *h* for each case of a rippled graphene sheet on a substrate

Rippled graphene on	*δ* (Å)	*ξ* (eV Å^−2^)	*h* (Å)
Cu(111) substrate	3.25^25^	0.02481^26^	8.0 δ
Ni(111) substrate	2.10^27^	0.09133^26^	6.7 δ

## 3 The transitional case

We now apply the calculus of variations to determine the conformation of a rippled graphene sheet and derive a solution for which the functional (1) is minimised. We impose an isoperimetric constraint on the total arc length of *C*, given by
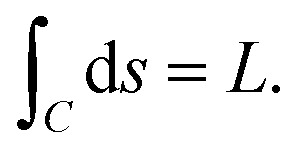


Moreover we assume that *x*_2_ is fixed, but *x*_1_ varies (to be determined from a natural boundary condition). Therefore the new functional we wish to minimise is of the form3
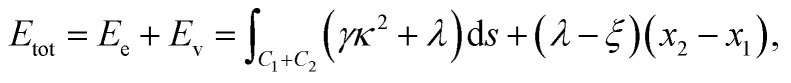
subject to the boundary conditions (2), where *λ* is a Lagrange multiplier corresponding to the isoperimetric constraint. We nondimensionalise (3) by defining *X* = *x*/*α* and *Y* = *y*/*α* where *α* is a scaling factor, and therefore *ÿ* = *Y*′′/*α* and *ẏ* = *Y*′, where primes denote derivatives with respect to the nondimensional *X* coordinate. The substitution of these new variables into eqn (3) yields4
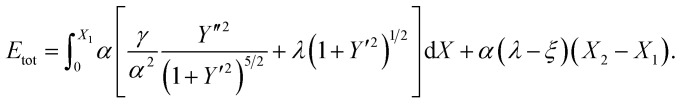


Furthermore we subtract the fixed energy at *X*_2_, and divide both sides of eqn (4) by *αξ*. Thus we derive the nondimensionalised energy functional as5
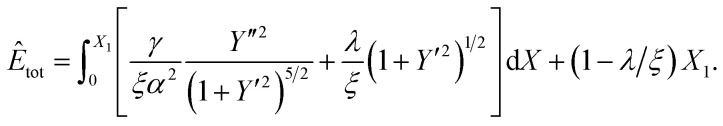


We let 
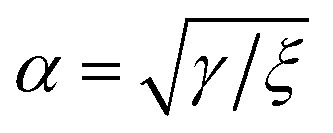
 and *μ* = *λ*/*ξ* so that6
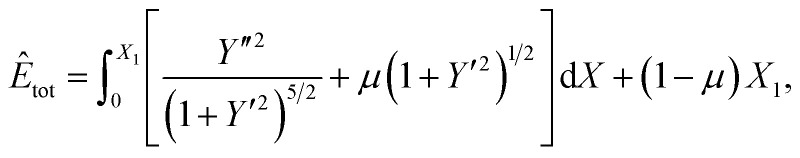
which is the functional we wish to minimise subject to the boundary condition *Y*(0) = *h*/*α* and a natural boundary condition applies at *X* = *X*_1_. To simplify the following calculations, we use *f* to denote the integrand part of (6), that is7
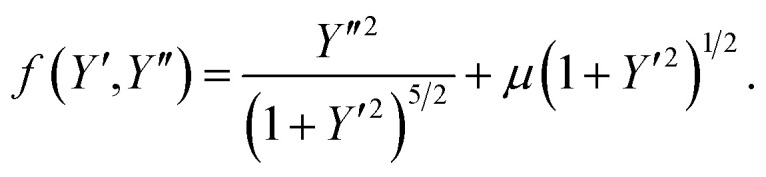


Extremals of (6) are given by Euler–Lagrange equation8
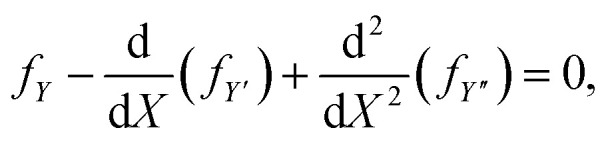
and since (7) does not depend on *Y* explicitly, then on integrating (8) with respect to *X*, we obtain the first integral9
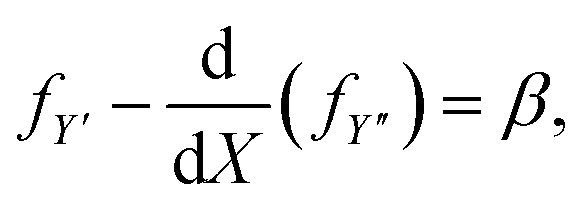
where *β* is a constant. Further, the integrand has no explicit dependence on *X*, this provides the additional first integral10
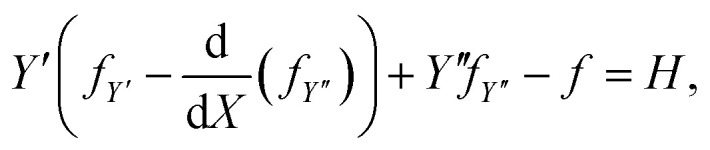
where *H* is a constant. On considering the corresponding second-order variational problem, we may derive the standard equation for the first variation of *Ê*_tot_ as
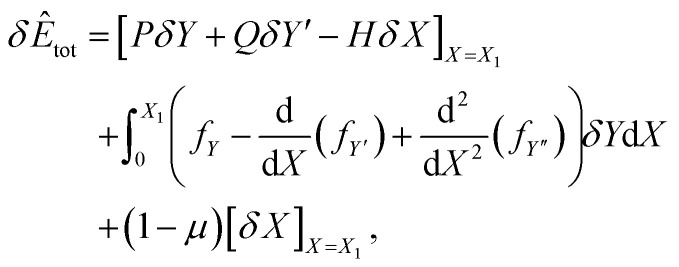
where *P* = *f*_*Y*′_ − d(*f*_*Y*′′_)/d*X*, *Q* = *f*_*Y*′′_, and *H* = (*Y*′*P* + *Y*′′*Q* − *f*). Here the natural boundary condition, which applies when the *X*-coordinate at the end point *X* = *X*_1_ is not prescribed, requires *H* = (1 − *μ*). Therefore by combining eqn (9) and (10), we obtain*βY*′ + *Y*′′*f*_*Y*′′_ − *f* = (1 − *μ*).

Which after the substitution of (7) leads to
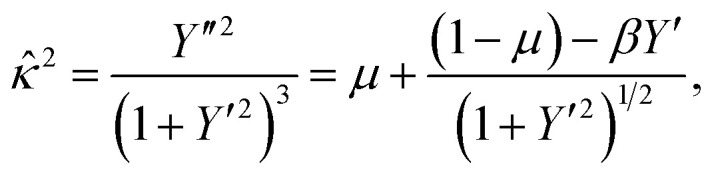
or equivalently
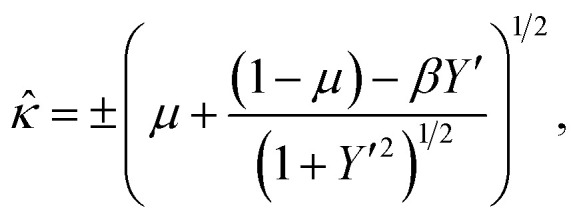
which is an equation relating the nondimensional curvature *κ̂* and the first derivative of *Y*. In order to obtain the parametric solutions, we make the substitution of *Y*′ = tan *θ* which yields*κ̂* = ±[*μ* + (1 − *μ*)cos *θ* − *β* sin *θ*]^1/2^.

The above expression may be written as follows
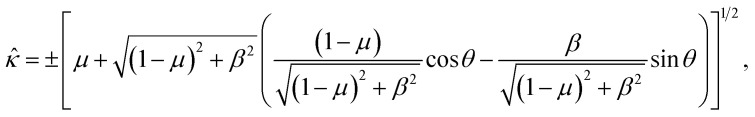
whereupon we define a new parameter *ψ*, such that
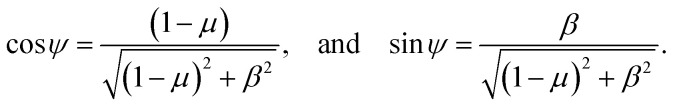


This leads to the succinct formula

11 *κ̂* = ±[*μ* + (1 − *μ*)sec *ψ* cos(*ψ* + *θ*)]^1/2^.

Now, using the fact that *κ̂* = cos(*θ*)d*θ*/d*X* = sin(*θ*)d*θ*/d*Y*, we derive two first order differential equations, namely12a
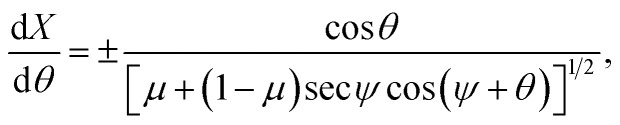
12b
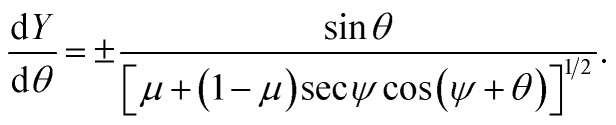


To facilitate further integration we change the parametric variable of our equations from *θ* to *ϕ* such that *θ* = 2*ϕ* − *ψ*, thus eqn (12a) and (12b) take the following forms13a
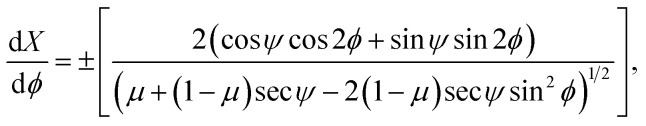
13b
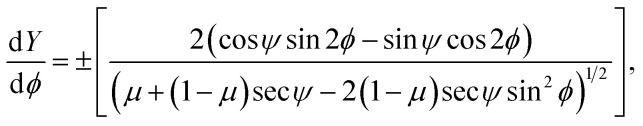
and we now obtain the general solutions by integrating eqn (13a) and (13b). This yields*X*(*ϕ*) = *c*_1_ ± *A*[cos *ψ* (*E*(*ϕ*,*k*) − *BF*(*ϕ*,*k*)) − sin *ψ* (1 − *k*^2^ sin^2^ *ϕ*)^1/2^],*Y*(*ϕ*) = *c*_2_ ∓ *A*[sin *ψ* (*E*(*ϕ*,*k*) − *BF*(*ϕ*,*k*)) + cos *ψ* (1 − *k*^2^ sin^2^ *ϕ*)^1/2^],with the parameters
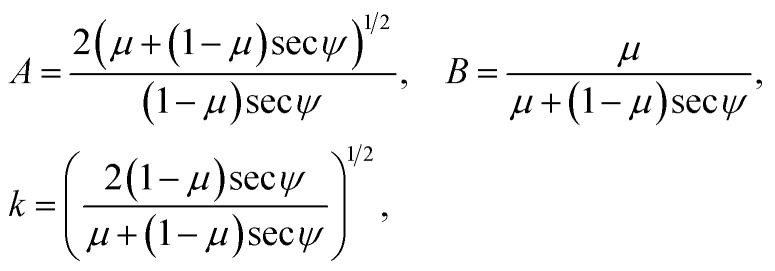
where *F*(*ϕ*,*k*) and *E*(*ϕ*,*k*) represent the incomplete elliptic integrals of the first and second kind, respectively, with elliptic modulus *k*, and *c*_1_, *c*_2_ are arbitrary constants of integration.

We now define two functions which play a significant role in determining our parametric solutions, namely

14a *g*_1_(*ϕ*) = *A*[(*E*(*ϕ*,*k*) − *E*(*ϕ*_0_,*k*)) − *B*(*F*(*ϕ*,*k*) − *F*(*ϕ*_0_,*k*))],

14b *g*_2_(*ϕ*) = *A*(1 − *k*^2^ sin^2^ *ϕ*)^1/2^.

The line curvature changes sign at *ϕ*_0_ = sin^−1^(1/*k*) corresponds to (*X*_0_, *Y*_0_) which is the boundary point between the two curves *C*_1_ and *C*_2_, which has coordinates

15a *X*_0_ = [sin *ψ g*_2_(*ψ*/2) − cos *ψ g*_1_(*ψ*/2)],

15b
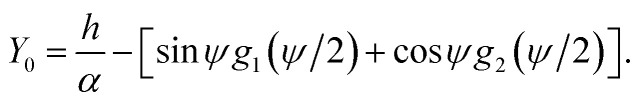


Moreover, the solutions are shown to have a rotational symmetry about this critical point (*X*_0_, *Y*_0_) where *ϕ* varies over the range [*ψ*/2, *ϕ*_0_]. Thus our solutions may be written in terms of *X*_0_ and *Y*_0_ as

16a *X*_*C*_1_/*C*_2__(*ϕ*) = *X*_0_ ± [cos *ψ g*_1_(*ϕ*) − sin *ψ g*_2_(*ϕ*)],

16b *Y*_*C*_1_/*C*_2__(*ϕ*) = *Y*_0_ ± [sin *ψ g*_1_(*ϕ*) + cos *ψ g*_2_(*ϕ*)].

Now, using the boundary condition *Y*_*C*_2__(*ψ*/2) = *δ*/*α*, we may obtain
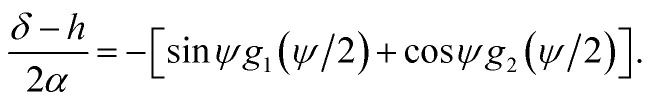


Hence we may rewrite eqn (15b) as
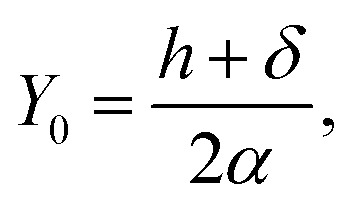
which introduces the *Y* component of the critical point as the midpoint of the ripple amplitude and the flat section of the graphene sheet. We also comment that *x* = *αX* and *y* = *αY*, and so multiplying these solutions by the scaling factor 
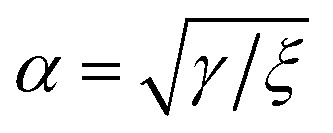
 recovers the re-dimensionalised solutions.

### Numerical results

**Fig. 3:**
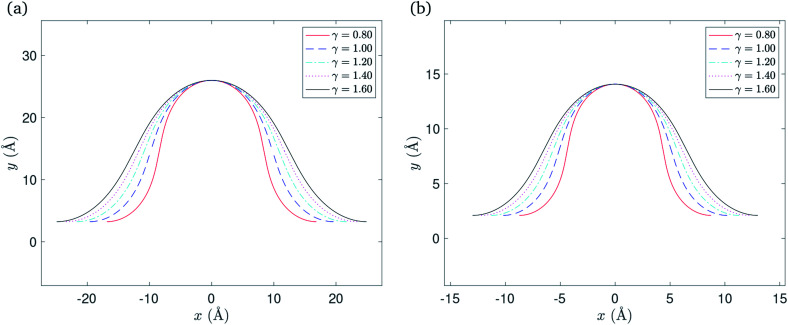
Rippled graphene sheet located on: (a) Cu(111) substrate and (b) Ni(111) substrate, for various bending rigidity *γ*, in the transitional case.

We are left with two parameters to determine, namely *ψ* and *μ*. We may find their values by solving the following system of equations numerically
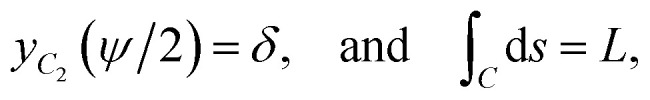
where *δ* takes a specific empirically determined value for each substrate as given in Table 1, and *L* is the assumed fixed arc length of *C*. After finding the unknowns *ψ* and *μ*, the solution is fully determined and representative plots are shown in Fig. 3. At this point, we may also give the total half arc length of the ripple *L*_rip_ by integrating d*s* over the curve *C*_1_ + *C*_2_,
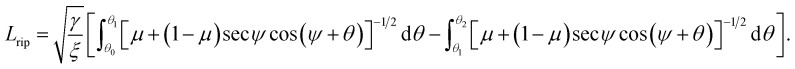


Again using the substitution of *θ* = 2*ϕ* − *ψ* we deduce that
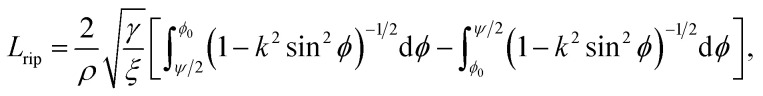
where 
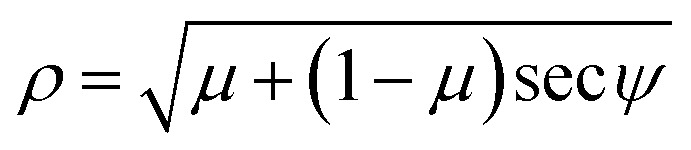
. Hence, the total half arc length of the ripple is given by17
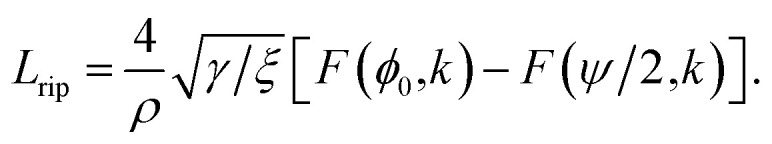


We may also calculate the elastic energy by integrating the square of the line curvature over the total length of *C*_1_ + *C*_2_, as follows
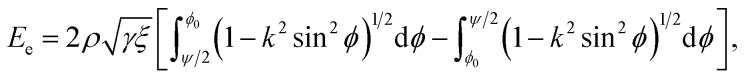
and therefore, the elastic energy is given by
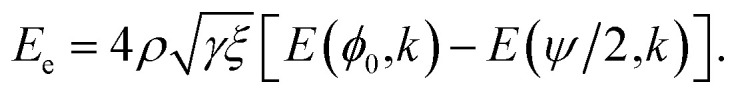


Hence, the total energy per unit length for this model is given by18
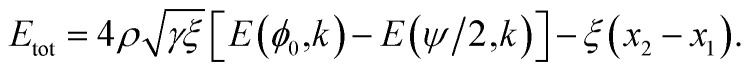


Numerical values for the *x*-component of the critical point *x*_0_ and the total half arc length of the ripple *L*_rip_ are presented in Table 2, for a range of values of the bending rigidity *γ*. The vdW interaction *ξ* takes a specific empirically derived value for each substrate as given in Table 1.

**Table 2:** The *x*-component of the critical point *x*_0_ and the ripple half arc length *L*_rip_ for various bending rigidities *γ*, in the transitional case

*γ* (eV)	Cu(111) substrate		Ni(111) substrate	
	*x*_0_ (Å)	*L*_rip_ (Å)	*x*_0_ (Å)	*L*_rip_ (Å)
0.80	8.42	30.86	4.36	16.17
1.00	9.80	32.29	5.10	16.92
1.20	10.84	33.52	5.65	17.57
1.40	11.71	34.61	6.11	18.14
1.60	12.45	35.59	6.50	18.65

## 4 The substrate constrained case

We now consider the special case, shown in Fig. 2(b), by removing the assumed isoperimetric constraint on the total arc length of *C* to allow graphene to overhang the substrate. Therefore we discard the variation of *x*_2_ and substitute *λ* = 0, and 
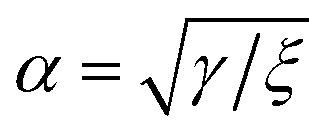
 into eqn (5) to obtain the functional that we wish to minimise, that is19
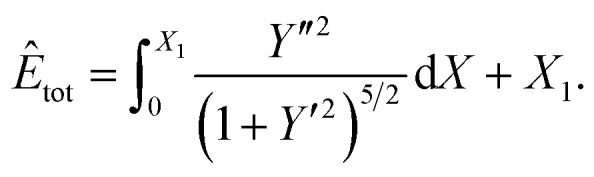


We note that this expression is identical to (6) with *μ* = 0, and so by the same reasoning as in Section 3 we obtain*κ̂* = ±[sec *ψ* cos(*ψ* + *θ*)]^1/2^.

Consequently, we redefine some of our parameters so that now *A* = 2(sec *ψ*)^−1/2^, *B* = 0, and 
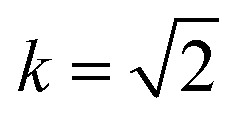
. Again the line curvature *κ̂* changes sign at *ϕ*_0_ = sin^−1^(1/*k*) = π/4 which corresponds to the point (*X*_0_, *Y*_0_) and has similar coordinates to that in (15a) and (15b). Taking the redefined parameters into account, these coordinates are*X*_0_ = [sin *ψ g*_2_(*ψ*/2) − cos *ψ g*_1_(*ψ*/2)],
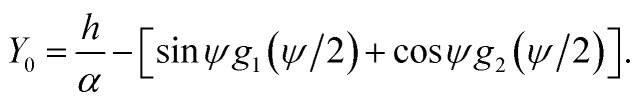


Similarly, with the rotational symmetry of the solutions about the point (*X*_0_, *Y*_0_), and the variation of *ϕ* over [*ψ*/2, π/4], the curves *C*_1_ and *C*_2_ still can be written as*X*_*C*_1_/*C*_2__(*ϕ*) = *X*_0_ ± [cos *ψ g*_1_(*ϕ*) − sin *ψ g*_2_(*ϕ*)],*Y*_*C*_1_/*C*_2__(*ϕ*)=*Y*_0_ ± [sin *ψ g*_1_(*ϕ*) + cos *ψ g*_2_(*ϕ*)].

Using the same derivation as in Section 3, the *Y* component of the critical point in this case may be rewritten as
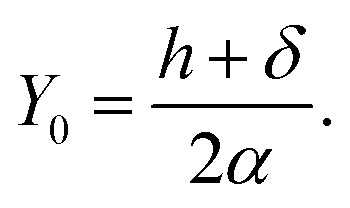


As before, multiplying the solutions above by the scaling factor 
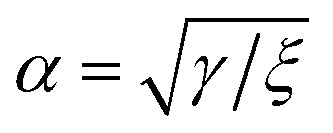
 recovers the dimensional solutions for the curves *C*_1_ and *C*_2_.

### Numerical results

**Fig. 4:**
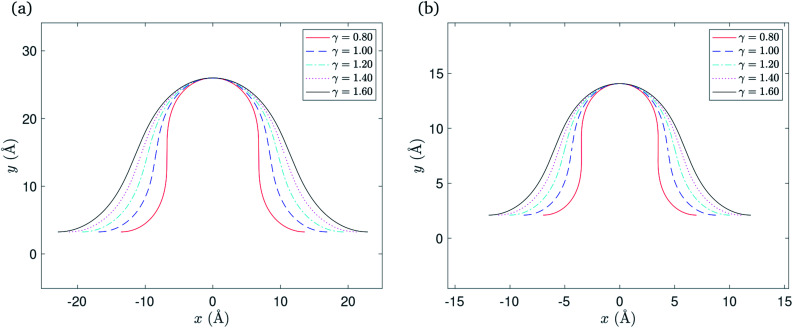
Rippled graphene sheet located on: (a) Cu(111) substrate and (b) Ni(111) substrate, for various bending rigidity *γ*, in the substrate constrained case.

In this case we have only one parameter to determine, namely *ψ*, and using the boundary condition *Y*_*C*_2__(*ψ*/2) = *δ*, the value of *ψ* may be determined numerically. After determining *ψ*, the solution is fully determined and representative plots are shown in Fig. 4. We comment here that the gradient of the graph for *γ* = 0.8 in the neighbourhood of the critical point becomes infinite when the ripple amplitude *h* ≈ 8.0* δ* for the Cu(111) substrate, and *h* ≈ 6.7* δ* for the Ni(111) substrate where *δ* takes a specific empirical value for each substrate as presented in Table 1. We note that by increasing the ripple height the graphene sheet starts to bend over itself which is the transition point from the rippled state to the wrinkled state. Also, higher bending rigidities require higher ripple amplitudes for the maximum gradient to approach infinity. However, we fix these values of *h* for each corresponding substrate in all plots to enable comparison between various values of the bending rigidity *γ*.

The total half arc length of the ripple *L*_rip_ and the total energy *E*_tot_ for this case can be calculated from eqn (17) and (18), respectively, with the new parameters *μ* = 0 and *ϕ*_0_ = π/4. Thus, the total half arc length of the ripple is given by
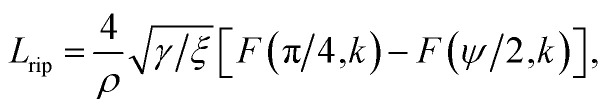
and the total energy per unit length is given by
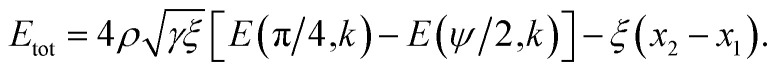


Numerical values for the *x*-component of the critical point *x*_0_ and the total half arc length of the ripple are shown in Table 3, for a range of values for the bending rigidity *γ*. Again the vdW interaction strength *ξ* takes a specific empirically derived value for each substrate as given in Table 1.

**Table 3:** The *x*-component of the critical point *x*_0_ and the ripple half arc length *L*_rip_ for various bending rigidities *γ*, in the substrate constrained case

*γ* (eV)	Cu(111) substrate		Ni(111) substrate	
	*x*_0_ (Å)	*L*_rip_ (Å)	*x*_0_ (Å)	*L*_rip_ (Å)
0.80	6.79	29.81	3.49	15.63
1.00	8.44	31.13	4.37	16.32
1.20	9.65	32.34	5.02	16.95
1.40	10.63	33.43	5.53	17.52
1.60	11.45	34.44	5.97	18.04

## 5 The graphene constrained case

In this case, as shown in Fig. 2(c), in addition to the assumed isoperimetric constraint on the total arc length of *C*, we take into account the variations of *x*_2_, and assume it varies proportionally with *x*_1_, that is
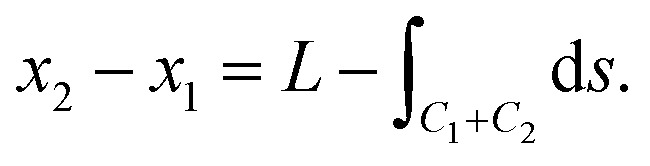


Following the nondimensionalisation described in Section 3, the total energy per unit length for this case is20
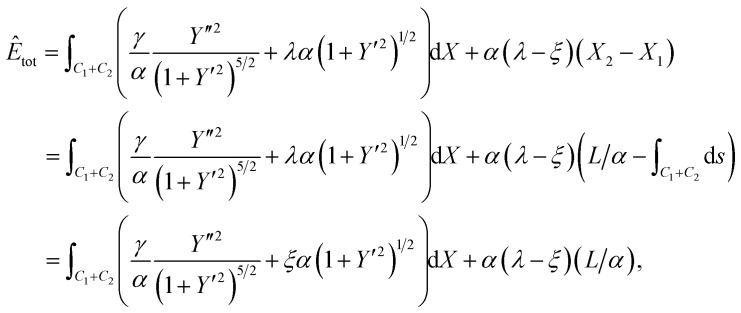
where d*s* = *α*(1 + *Y*′^2^)^1/2^d*X*. We now divide both sides by *ξα* and set 
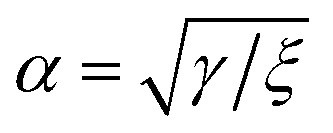
. Further, the aim is to determine the shape of *Y* = *Y*(*X*) which minimises *Ê*_tot_, and here *λ* has no impact on the solution, so we choose *λ* = *ξ* to simplify the calculation. The final form of the functional to be minimised is21
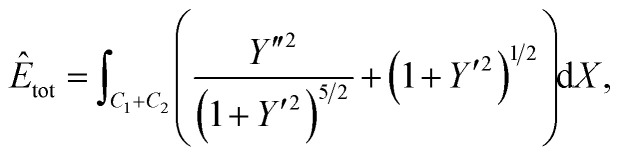
subject to the boundary conditions provided in Section 2. Comparing (21) and (6), we find that *μ* = 1. In a similar way as in the previous two cases, the use of calculus of variations then provides that

22 *κ̂* = ±[1 − *β* sin *θ*]^1/2^.

Similarly, by comparing (22) with the equivalent expression for the transitional case (11), we note that in (22)*ψ* = π/2 and *β* acts in place of the term 
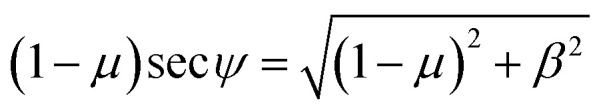
 as *μ* approaches 1. Therefore, we redefine the parameters *A*, *B*, and *k* in (14a) and (14b) as
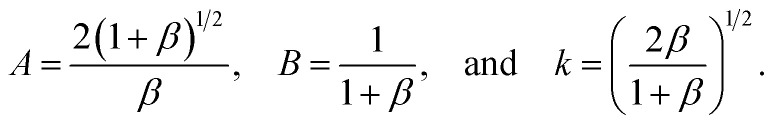


The critical point (*X*_0_, *Y*_0_) represents the point of the rotational symmetry which again corresponds to *ϕ*_0_ = sin^−1^(1/*k*), and the coordinates of this point are given by
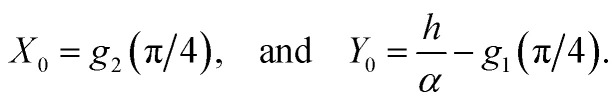


Similarly, the solutions on the curves *C*_1_ and *C*_2_ where *ϕ* ∈ [*ϕ*_0_, π/4] are*X*_*C*_1_/*C*_2__(*ϕ*) = *X*_0_ ∓ *g*_2_(*ϕ*), and *Y*_*C*_1_/*C*_2__(*ϕ*) = *Y*_0_ ± *g*_1_(*ϕ*).

Now, we use the boundary condition *Y*_*C*_2__(π/4) = *δ*/*α* to rewrite the *Y* component of the critical point as
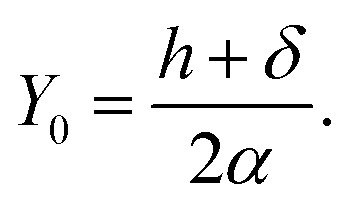


The dimensional solutions for the curves *C*_1_ and *C*_2_ may be obtained by scaling these solution by 
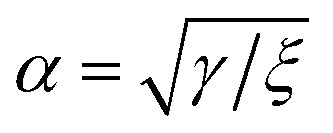
.

### Numerical results

**Fig. 5:**
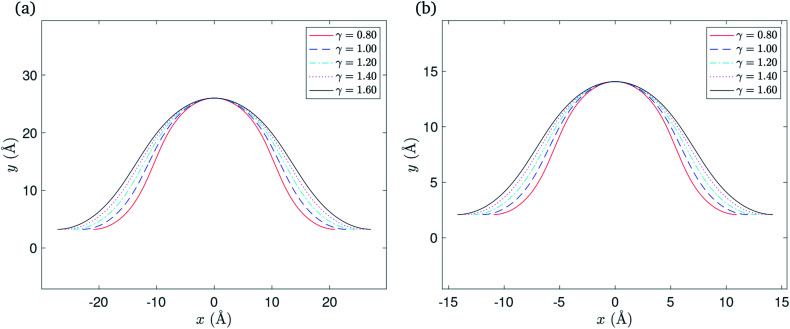
Rippled graphene sheet located on: (a) Cu(111) substrate and (b) Ni(111) substrate, for various bending rigidity *γ*, in the graphene constrained case.

Here again we are left with only one parameter to determine, namely *β*, and using the boundary condition *Y*_*C*_2__(π/4) = *δ*, the value of *β* may be determined numerically. After finding *β*, the solution is fully determined and representative plots are shown in Fig. 5. The total half arc length of the ripple *L*_rip_ for this case can be evaluated from (17) with *ψ* = π/2 and 
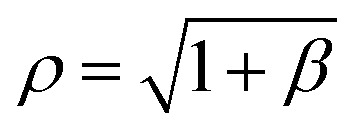
 to give
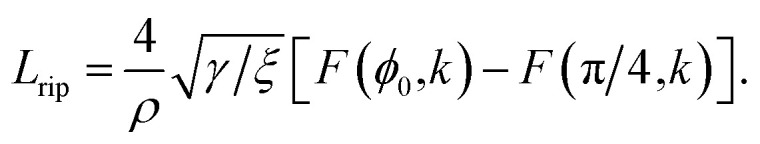


Similarly, the total energy per unit length *E*_tot_ for this case is obtained from (18), which yields
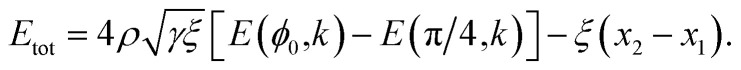


Numerical values for the *x*-component of the critical point *x*_0_ and the total half arc length of the ripple *L*_rip_ are presented in Table 4 for a range of values of the bending rigidity *γ*. As before, the vdW interaction *ξ* takes a specific empirically derived value for each substrate as given in Table 1.

**Table 4:** The *x*-component of the critical point *x*_0_ and the ripple half arc length *L*_rip_ for various bending rigidities *γ*, in the graphene constrained case

*γ* (eV)	Cu(111) substrate		Ni(111) substrate	
	*x*_0_ (Å)	*L*_rip_ (Å)	*x*_0_ (Å)	*L*_rip_ (Å)
0.80	10.47	32.76	5.47	17.18
1.00	11.43	34.00	5.97	17.83
1.20	12.24	35.11	6.39	18.41
1.40	12.95	36.11	6.77	18.93
1.60	13.59	37.02	7.10	19.41

## 6 Results

For the purpose of comparison, we assume a fixed half length *L* = 700 Å for the rippled graphene sheet on a flat substrate. The goal is to obtain a relationship between the variation of the substrate length and the total energy per unit length for the rippled graphene. The substrate half length *L*_sub_ is divided into three regimes that correspond to the point *x*_2_, where the graphene edge ends, which can be calculated by *x*_2_ = *x*_1_ + *L* − *L*_rip_. Here we comment that *x*_2s_ < *x*_2g_ where *x*_2s_ and *x*_2g_ correspond to the substrate constrained case and the graphene constrained case, respectively.

**Fig. 6:**
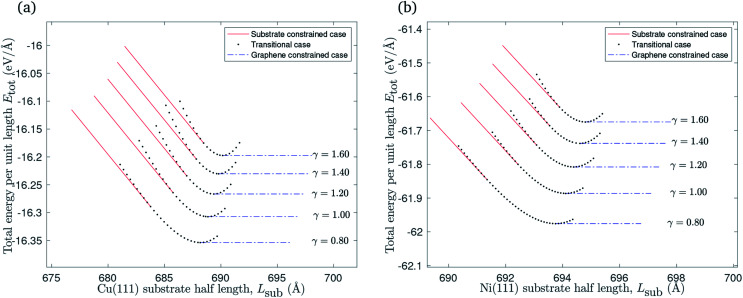
The relationship between varying the substrate length of: (a) Cu(111) substrate, (b) Ni(111) substrate and the total energy per unit length.

The substrate constrained case was formulated to describe the regime where *L*_sub_ < *x*_2s_, provided that as *L*_sub_ gets closer to *x*_2s_ the total energy per unit length *E*_tot_ decreases due to the vdW interactions that occur when *L*_sub_ increases. The graphene constrained case can be adapted to address the regime when *L*_sub_ > *x*_2g_, and here the total energy per unit length *E*_tot_ remains constant since there are no more interactions that are involved for this section. Finally, the transitional case is used to evaluate the total energy per unit length *E*_tot_ when *L*_sub_ ≈ *x*_2_. Multiple values for *x*_2t_ were chosen from the interval [*x*_2s_, *x*_2g_] to fill the gap between the substrate constrained case and the graphene constrained case where *x*_2t_ denotes the location of the graphene edge in the transitional case. Fig. 6 illustrates the relationship between varying the substrate length and total energy per unit length included the three cases discussed above.

**Fig. 7:**
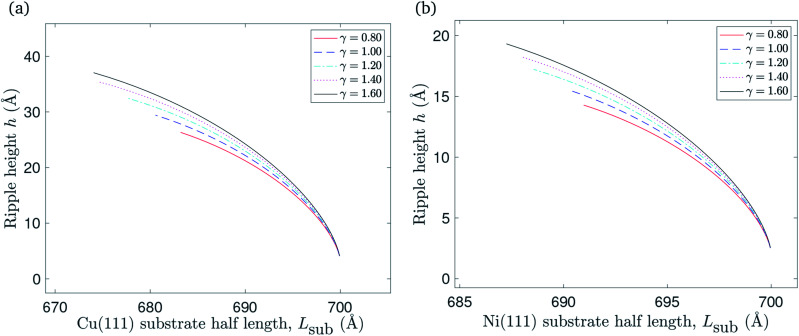
The relationship between reducing the substrate length of: (a) Cu(111) substrate, (b) Ni(111) substrate and the ripple amplitude.

A graphene sheet of half length *L* = 700 Å will remain flat when it is placed on a flat substrate of the same half length. The substrate constrained case can be adapted to investigate the impact of reducing the substrate length on the graphene sheet. Fig. 7 shows that ripples form in this scenario, and that there is an increase in the ripple amplitude as the substrate length decreases. Graphene of lower bending rigidity reaches the ripple height for which the gradient of the ripple becomes infinite at a relatively longer substrate than graphene with a higher bending rigidity. The substrate length at which the ripple gradient becomes infinite corresponds to the left end-point of each curve in Fig. 7.

**Fig. 8:**
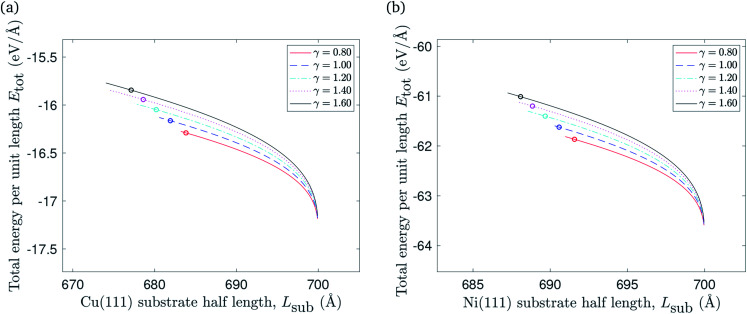
The relationship between reducing the substrate length of: (a) Cu(111) substrate, (b) Ni(111) substrate and the total energy per unit length.

The impact of reducing the substrate length on the total energy per unit length is presented in Fig. 8. The total energy per unit length of the flat graphene sheet is approximated at −17.2 eV Å^−1^ when it is placed on copper, and at −63.5 eV Å^−1^ in the case of a nickel substrate. It increases when the substrate length decreases, as expected due to reduced vdW interactions between the graphene sheet and the substrate, as well as elastic energy stored in the ripple. The points marked with circles represent (*L*_sub_0__, *E*_tot_(*L*_sub_0__)) where *L*_sub_0__ denotes the substrate length when the gradient of the total energy per unit length curve approaches −*ξ*.

Starting from a flat graphene sheet laying on a shrinking substrate, the system might form a ripple or remain flat and beginning to overhang the substrate. The ripple formation pathway would follow the transitional case and the flat pathway would follow the substrate constrained case. In an ideal setting, ripples would not form because the energy require to follow the first pathway exceeds the energy lost in following the second. However, we comment that many experimental situations include confounding effects such as impurities and defects in the graphene structure which are not accounted for in this work.

**Fig. 9:**
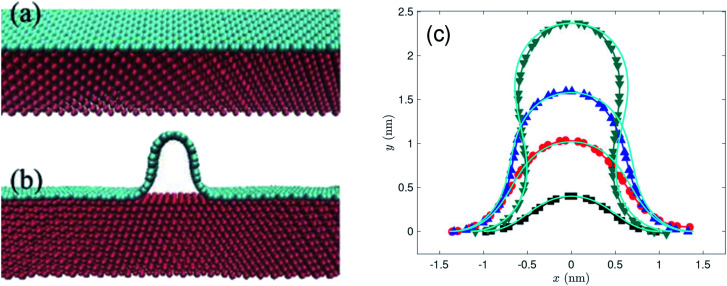
MD simulation models and results of graphene wrinkles reprinted (or adapted) from ref. 28, licensed under a Creative Commons Attribution 4.0 International License. (a and b) The MD simulation model, performed by Wang *et al.*,^28^ is presented here in order to adopt the appropriate case among those developed in this model for comparison purposes. (c) The profiles of graphene ripples or wrinkles obtained by present model (cyan lines) superimposed upon the results of MD simulation.^28^

Recently, morphologies of graphene wrinkles on copper substrate were experimentally investigated by Wang *et al.*^28^ They observed unexpected profiles of wrinkles with widths in the range of tens of nanometres, and heights in the range of a few nanometres. Their theoretical methods and MD simulations agree that the maximum width of wrinkles is less than 2.7 nm which is largely smaller than those experimentally observed. As they reported, the presence of interlayer molecules between the graphene and the copper substrate is the main mechanism that enlarges both width and height of graphene wrinkle. Since we don't take into account interlayer molecules, our model is comparable with their MD simulations without interlayer molecules. Their MD simulation model, as reprinted in Fig. 9(a) and (b), is a stack of two rectangular materials, upper cyan graphene and lower red copper which is best modelled by the transitional case in our model. Therefore, the conformations of rippled (or wrinkled) graphene on a copper substrate, obtained by the transitional case, with bending rigidity *γ* = 1.20 eV and different heights are adopted to compare with the results of MD simulations of graphene wrinkles obtained by Wang *et al.*,^28^ as shown in Fig. 9(c). Despite the fact that the present model uses the value of 2.481 eV nm^−2^ for the vdW energy of graphene–Cu(111) interactions per unit area, but their model used the value of 4.494 eV nm^−2^, both results are in excellent agreement in predicting the profiles of graphene ripples (or wrinkles).

## 7 Summary

In this paper, we develop a model for a rippled graphene sheet that is located on a flat metal substrate. The assumed translational symmetry along the ripple reduces our problem to two dimensions, and the reflective symmetry about the *y*-axis allows us to consider only solutions in the upper right quadrant. We employ variational calculus to minimise the elastic energy arising from the curvature squared and maximising the vdW interaction energy between the flat section of the graphene and the metal substrate.

We account for the length of the substrate by considering three different cases. The first addresses the case when the edges of the rippled graphene sheet and the substrate coincide. The second case is when the edge of the graphene sheet overhangs the substrate edge, and the last case applies to a rippled graphene sheet for which the edge of the sheet does not extend to the substrate edge. We assume a fixed half length of the graphene sheet, and consider all three cases to obtain a continuous relationship between the total energy per unit length of the graphene and the length of the substrate as shown in Fig. 6. The substrate constrained case is used to illustrate the effect of reducing the substrate length on the ripple height and the total energy of the graphene as shown in Fig. 7 and 8. Using the transitional case, our model is shown to agree with the results of earlier MD simulations of graphene ripples (or wrinkles) on a Cu(111) substrate.

Future research may address the phenomenon of ripple formation when the substrate shrinks by taking into account additional physical effects. For instance the model maybe extended to account for the change in the vdW interaction strength as the substrate length and density change. This will introduce *ξ* as a function of *x* and lead to an explicit dependence on *x* in the integrand part of (6), which would undoubtedly complicate the corresponding Euler–Lagrange equation.

## Conflicts of interest

There are no conflicts to declare.

## Supplementary Material
